# Tetra-μ_3_-*tert*-butano­lato-tetra­thallium(I)

**DOI:** 10.1107/S1600536810047550

**Published:** 2010-11-20

**Authors:** Florian Blasberg, Hans-Wolfram Lerner, Michael Bolte

**Affiliations:** a Institut für Anorganische Chemie, J. W. Goethe-Universität Frankfurt, Max-von-Laue-Strasse 7, 60438 Frankfurt/Main, Germany

## Abstract

The title compound, [Tl_4_(C_4_H_9_O)_4_], featuring a (Tl—O)_4_ cube, crystallizes with a quarter-mol­ecule (located on a special position of site symmetry 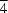..) and a half-mol­ecule (located on a special position of site symmetry 23.) in the asymmetric unit. The Tl—O bond distances range from 2.463 (12) to 2.506 (12) Å. All O—Tl—O bond angles are smaller than 90° whereas the Tl—O—Tl angles are wider than a recta­ngular angle.

## Related literature

For the use of bulky silyl chalcogenolate ligands of the type *E*Si*R*_3_^−^ and alkyl chalcogenolates *E*(alk­yl)^−^ (*E* = O, S, Se, Te) with especially bulky alkoxides to stabilize transition metal centres, see: Wolczanski (2009 ▶); Kückmann *et al.* (2005 ▶, 2008 ▶, 2010 ▶). For substitution reactions of transition metal atoms, see: Kern *et al.* (2008 ▶); Lerner *et al.* (2002 ▶, 2005 ▶). The title compound was prepared according to a slightly changed published procedure, see: Schmidbaur *et al.* (1968 ▶).
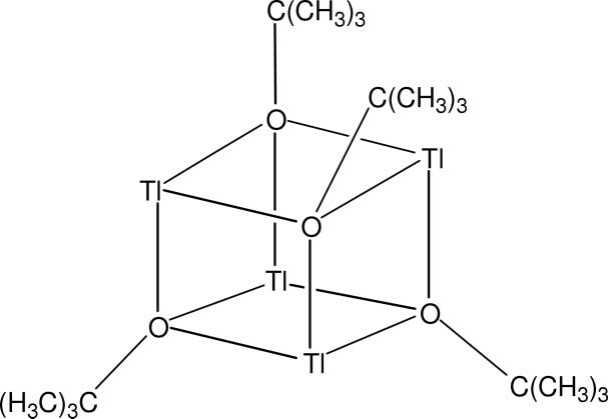


## Experimental

### 

#### Crystal data

[Tl_4_(C_4_H_9_O)_4_]
*M* *_r_* = 1109.93
Cubic, 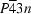

*a* = 17.1500 (15) Å
*V* = 5044.2 (8) Å^3^
*Z* = 8
Mo *K*α radiation
μ = 25.49 mm^−1^
*T* = 173 K
0.21 × 0.18 × 0.10 mm

#### Data collection

Stoe IPDS II two-circle diffractometer
Absorption correction: multi-scan (*MULABS*; Spek, 2009 ▶; Blessing, 1995 ▶) *T*_min_ = 0.075, *T*_max_ = 0.185
13612 measured reflections
1489 independent reflections
1226 reflections with *I* > 2σ(*I*)
*R*_int_ = 0.084

#### Refinement

*R*[*F* ^2^ > 2σ(*F* ^2^)] = 0.042
*wR*(*F* ^2^) = 0.083
*S* = 1.00
1489 reflections
73 parameters
6 restraints
H-atom parameters constrained
Δρ_max_ = 1.77 e Å^−3^
Δρ_min_ = −1.01 e Å^−3^
Absolute structure: Flack (1983 ▶), 711 Friedel pairs
Flack parameter: 0.00 (7)

### 

Data collection: *X-AREA* (Stoe & Cie, 2001 ▶); cell refinement: *X-AREA*; data reduction: *X-AREA*; program(s) used to solve structure: *SHELXS97* (Sheldrick, 2008 ▶); program(s) used to refine structure: *SHELXL97* (Sheldrick, 2008 ▶); molecular graphics: *XP* (Sheldrick, 2008 ▶); software used to prepare material for publication: *SHELXL97*.

## Supplementary Material

Crystal structure: contains datablocks I, global. DOI: 10.1107/S1600536810047550/kj2165sup1.cif

Structure factors: contains datablocks I. DOI: 10.1107/S1600536810047550/kj2165Isup2.hkl

Additional supplementary materials:  crystallographic information; 3D view; checkCIF report

## Figures and Tables

**Fig. 1.**
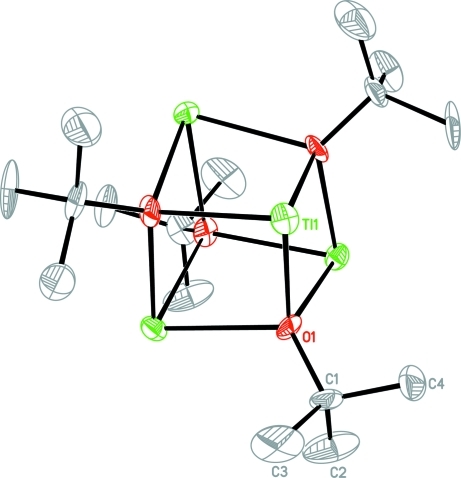
Perspective view of one of the two independent molecules of the title compound with the atom numbering scheme for the symmetry independent atoms; displacement ellipsoids are at the 50% probability level; H atoms are omitted for clarity.

**Fig. 2.**
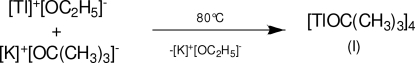
Preparation of the title compound.

## supplementary crystallographic information

### Comment

In a number of recent studies bulky silyl chalcogenolate ligands of the type ESi*R*_3_^-^ and alkyl chalcogenolates *E*(alkyl)^-^ (E = O, S, Se, Te) with especially bulky alkoxides have been used to stabilize transition metal centers (Wolczanski, 2009; Kückmann *et al.* 2005, 2008, 2010). In macromolecular chemistry, these ligands have also found application. Chalcogen-based ligands offer a variety of possible binding modes. Chalcogenolates are often found bridging two or more metal ions. Normally, transition metal complexes possess 6 e^-^ thiolate ligands in a µ_3_-binding mode. Recently, however, we have shown that the anion of the mixed-valence Mn(I/II) complex Na(thf)_6_[(OC)_3_Mn(µ-SSi*t*Bu_3_)_3_MnSSitBu_3_] contains a terminal thiolate ligand with a linear Mn—S—Si unit (Kückmann *et al.* 2008). The prerequisite for six-electron donation (2 σ- and 4 π-electrons) comparable with Cp^-^ is thus fulfilled. One approach is to create such complexes by substitution reactions of transition metal halogenides with alkali metal alkoxides as *M*^+^[OC(CH_3_)_3_]^-^ or alkali metal siloxides *M*^+^[OSi*R*_3_]- (Kern *et al.* 2008; Lerner *et al.* 2005, 2002). In most cases the reactions that occur between alkali metal alkoxides and transition metal halides are not quantitative. Another approach to complexes with chalcogen coordination is to start from thallium alkoxides which react almost quantitatively with transition metal chlorides due to the poor solubility of TlCl. In this paper we report the synthesis and the crystal structure of [TlO*t*Bu]_4_. The title compound [TlO*t*Bu]_4_ was prepared according to a slightly changed published procedure (Schmidbaur *et al.* 1968), as shown in Fig. 2. The following modifications have been made in our approach: thallium ethoxide was used instead of thallium methoxide and potassium *tert*-butoxide was substituted for sodium *tert*-butoxide.

### Experimental

In a flame-dried vial 1.1 ml thallium ethoxide (3.77 g, 15.1 mmol) was added to 1.70 g potassium *tert*-butoxide (15.1 mmol) in 50 ml benzene. After flame-sealing, the vial was heated to 80 °C for four days. The vial was opened, the crude reaction mixture filtered hot under an nitrogen atmosphere, the solid residue was washed with 20 ml benzene and the combined filtrates evaporated to dryness. The remaining colorless solid was suspended in ether and allowed to settle. A sample of the supernatant was transferred to a flame-dried Schlenk vessel and stored at -35°C. After two days colorless crystals of the composition [TlO*t*Bu]_4_ deposited and were separated from the mother liquor (Yield 15%).

### Refinement

H atoms were located in a difference map, but geometrically positioned and refined using a riding model with fixed individual displacement parameters [U(H) = 1.5 *U*_eq_(C)] and with C—H = 0.98 Å.

### Crystal data

**Table:**

[Tl_4_(C_4_H_9_O)_4_]	*D*_x_ = 2.923 Mg m^−^^3^
*M**_r_* = 1109.93	Mo *K*α radiation, λ = 0.71073 Å
Cubic, *P*43*n*	Cell parameters from 7201 reflections
Hall symbol: P -4n 2 3	θ = 3.4–25.9°
*a* = 17.1500 (15) Å	µ = 25.49 mm^−^^1^
*V* = 5044.2 (8) Å^3^	*T* = 173 K
*Z* = 8	Plate, colourless
*F*(000) = 3904	0.21 × 0.18 × 0.10 mm

### Data collection

**Table:**

Stoe IPDS II two-circle diffractometer	1489 independent reflections
Radiation source: fine-focus sealed tube	1226 reflections with *I* > 2σ(*I*)
graphite	*R*_int_ = 0.084
ω scans	θ_max_ = 25.0°, θ_min_ = 3.4°
Absorption correction: multi-scan (*MULABS*; Spek, 2009; Blessing, 1995)	*h* = −18→20
*T*_min_ = 0.075, *T*_max_ = 0.185	*k* = −19→20
13612 measured reflections	*l* = −20→18

### Refinement

**Table:**

Refinement on *F*^2^	Secondary atom site location: difference Fourier map
Least-squares matrix: full	Hydrogen site location: inferred from neighbouring sites
*R*[*F*^2^ > 2σ(*F*^2^)] = 0.042	H-atom parameters constrained
*wR*(*F*^2^) = 0.083	*w* = 1/[σ^2^(*F*_o_^2^) + (0.037*P*)^2^] where *P* = (*F*_o_^2^ + 2*F*_c_^2^)/3
*S* = 1.00	(Δ/σ)_max_ = 0.001
1489 reflections	Δρ_max_ = 1.77 e Å^−^^3^
73 parameters	Δρ_min_ = −1.01 e Å^−^^3^
6 restraints	Absolute structure: Flack (1983), 711 Friedel pairs
Primary atom site location: structure-invariant direct methods	Flack parameter: 0.00 (7)

### Special details

**Table:**

Geometry. All e.s.d.'s (except the e.s.d. in the dihedral angle between two l.s. planes) are estimated using the full covariance matrix. The cell e.s.d.'s are taken into account individually in the estimation of e.s.d.'s in distances, angles and torsion angles; correlations between e.s.d.'s in cell parameters are only used when they are defined by crystal symmetry. An approximate (isotropic) treatment of cell e.s.d.'s is used for estimating e.s.d.'s involving l.s. planes.
Refinement. Refinement of *F*^2^ against ALL reflections. The weighted *R*-factor *wR* and goodness of fit *S* are based on *F*^2^, conventional *R*-factors *R* are based on *F*, with *F* set to zero for negative *F*^2^. The threshold expression of *F*^2^ > σ(*F*^2^) is used only for calculating *R*-factors(gt) *etc*. and is not relevant to the choice of reflections for refinement. *R*-factors based on *F*^2^ are statistically about twice as large as those based on *F*, and *R*- factors based on ALL data will be even larger.

### Fractional atomic coordinates and isotropic or equivalent isotropic displacement parameters (Å^2^)

**Table:**

	*x*	*y*	*z*	*U*_iso_*/*U*_eq_	
Tl1	0.66760 (4)	0.91953 (4)	0.57229 (3)	0.02168 (17)	
O1	0.8130 (7)	0.9310 (7)	0.5635 (7)	0.022 (2)	
C1	0.8550 (13)	0.8777 (10)	0.6115 (13)	0.029 (4)	
C2	0.9386 (12)	0.8950 (18)	0.6078 (19)	0.059 (8)	
H2A	0.9478	0.9485	0.6256	0.088*	
H2B	0.9569	0.8895	0.5539	0.088*	
H2C	0.9671	0.8586	0.6414	0.088*	
C3	0.8400 (19)	0.7958 (10)	0.583 (2)	0.059 (7)	
H3A	0.7839	0.7853	0.5844	0.089*	
H3B	0.8669	0.7588	0.6177	0.089*	
H3C	0.8596	0.7901	0.5301	0.089*	
C4	0.8261 (16)	0.8852 (14)	0.6961 (12)	0.041 (6)	
H4A	0.8368	0.9380	0.7153	0.061*	
H4B	0.8533	0.8472	0.7290	0.061*	
H4C	0.7698	0.8753	0.6980	0.061*	
Tl1A	0.42129 (4)	0.42129 (4)	0.42129 (4)	0.0223 (2)	
O1A	0.5652 (6)	0.4348 (6)	0.4348 (6)	0.020 (4)	
C1A	0.6118 (13)	0.3882 (13)	0.3882 (13)	0.020 (7)	
C2A	0.6960 (12)	0.3942 (13)	0.4110 (14)	0.035 (5)	
H2A1	0.7125	0.4488	0.4086	0.053*	
H2A2	0.7277	0.3631	0.3751	0.053*	
H2A3	0.7027	0.3746	0.4643	0.053*	

### Atomic displacement parameters (Å^2^)

**Table:**

	*U*^11^	*U*^22^	*U*^33^	*U*^12^	*U*^13^	*U*^23^
Tl1	0.0231 (3)	0.0211 (3)	0.0208 (3)	−0.0047 (3)	0.0036 (3)	0.0011 (3)
O1	0.031 (6)	0.020 (6)	0.015 (6)	0.005 (5)	−0.005 (5)	0.009 (5)
C1	0.036 (10)	0.016 (9)	0.034 (12)	0.012 (8)	−0.009 (9)	0.003 (8)
C2	0.023 (10)	0.065 (16)	0.09 (2)	0.006 (12)	0.006 (13)	0.040 (14)
C3	0.09 (2)	0.007 (8)	0.08 (2)	0.007 (11)	−0.018 (18)	0.008 (12)
C4	0.063 (16)	0.041 (13)	0.019 (10)	0.018 (11)	0.007 (10)	0.003 (8)
Tl1A	0.0223 (2)	0.0223 (2)	0.0223 (2)	−0.0035 (3)	−0.0035 (3)	−0.0035 (3)
O1A	0.020 (4)	0.020 (4)	0.020 (4)	0.005 (5)	0.005 (5)	−0.005 (5)
C1A	0.020 (7)	0.020 (7)	0.020 (7)	0.003 (8)	0.003 (8)	−0.003 (8)
C2A	0.025 (8)	0.041 (8)	0.041 (9)	0.011 (6)	0.007 (7)	−0.010 (7)

### Geometric parameters (Å, °)

**Table:**

Tl1—O1^i^	2.463 (12)	C4—H4A	0.9800
Tl1—O1^ii^	2.493 (11)	C4—H4B	0.9800
Tl1—O1	2.506 (12)	C4—H4C	0.9800
O1—C1	1.42 (2)	Tl1A—O1A	2.490 (8)
O1—Tl1^ii^	2.463 (12)	Tl1A—O1A^iii^	2.490 (8)
O1—Tl1^i^	2.492 (11)	Tl1A—O1A^iv^	2.490 (8)
C1—C2	1.47 (3)	O1A—C1A	1.38 (4)
C1—C3	1.51 (3)	O1A—Tl1A^iii^	2.490 (8)
C1—C4	1.54 (3)	O1A—Tl1A^iv^	2.490 (8)
C2—H2A	0.9800	C1A—C2A^v^	1.50 (2)
C2—H2B	0.9800	C1A—C2A^vi^	1.50 (2)
C2—H2C	0.9800	C1A—C2A	1.50 (2)
C3—H3A	0.9800	C2A—H2A1	0.9800
C3—H3B	0.9800	C2A—H2A2	0.9800
C3—H3C	0.9800	C2A—H2A3	0.9800
			
O1^i^—Tl1—O1^ii^	81.0 (4)	H3B—C3—H3C	109.5
O1^i^—Tl1—O1	78.3 (4)	C1—C4—H4A	109.5
O1^ii^—Tl1—O1	77.8 (4)	C1—C4—H4B	109.5
O1^i^—Tl1—Tl1^vii^	41.8 (3)	H4A—C4—H4B	109.5
O1^ii^—Tl1—Tl1^vii^	41.2 (3)	C1—C4—H4C	109.5
O1—Tl1—Tl1^vii^	84.3 (2)	H4A—C4—H4C	109.5
C1—O1—Tl1^ii^	119.7 (10)	H4B—C4—H4C	109.5
C1—O1—Tl1^i^	119.3 (11)	O1A—Tl1A—O1A^iii^	78.9 (6)
Tl1^ii^—O1—Tl1^i^	97.0 (4)	O1A—Tl1A—O1A^iv^	78.9 (6)
C1—O1—Tl1	114.7 (11)	O1A^iii^—Tl1A—O1A^iv^	78.9 (6)
Tl1^ii^—O1—Tl1	101.8 (4)	C1A—O1A—Tl1A^iii^	117.7 (11)
Tl1^i^—O1—Tl1	101.0 (4)	C1A—O1A—Tl1A^iv^	117.7 (11)
O1—C1—C2	109.8 (17)	Tl1A^iii^—O1A—Tl1A^iv^	100.1 (5)
O1—C1—C3	109.2 (17)	C1A—O1A—Tl1A	117.7 (11)
C2—C1—C3	110 (2)	Tl1A^iii^—O1A—Tl1A	100.1 (5)
O1—C1—C4	109.1 (16)	Tl1A^iv^—O1A—Tl1A	100.1 (5)
C2—C1—C4	110 (2)	O1A—C1A—C2A^v^	111.4 (15)
C3—C1—C4	109 (2)	O1A—C1A—C2A^vi^	111.4 (15)
C1—C2—H2A	109.5	C2A^v^—C1A—C2A^vi^	107.4 (16)
C1—C2—H2B	109.5	O1A—C1A—C2A	111.4 (15)
H2A—C2—H2B	109.5	C2A^v^—C1A—C2A	107.4 (16)
C1—C2—H2C	109.5	C2A^vi^—C1A—C2A	107.4 (16)
H2A—C2—H2C	109.5	C1A—C2A—H2A1	109.5
H2B—C2—H2C	109.5	C1A—C2A—H2A2	109.5
C1—C3—H3A	109.5	H2A1—C2A—H2A2	109.5
C1—C3—H3B	109.5	C1A—C2A—H2A3	109.5
H3A—C3—H3B	109.5	H2A1—C2A—H2A3	109.5
C1—C3—H3C	109.5	H2A2—C2A—H2A3	109.5
H3A—C3—H3C	109.5		
			
O1^i^—Tl1—O1—C1	−137.9 (13)	Tl1—O1—C1—C4	−53.8 (18)
O1^ii^—Tl1—O1—C1	139.0 (13)	O1A^iii^—Tl1A—O1A—C1A	139.7 (13)
Tl1^vii^—Tl1—O1—C1	−179.8 (12)	O1A^iv^—Tl1A—O1A—C1A	−139.7 (13)
O1^i^—Tl1—O1—Tl1^ii^	91.4 (5)	O1A^iii^—Tl1A—O1A—Tl1A^iii^	10.8 (5)
O1^ii^—Tl1—O1—Tl1^ii^	8.2 (4)	O1A^iv^—Tl1A—O1A—Tl1A^iii^	91.49 (15)
Tl1^vii^—Tl1—O1—Tl1^ii^	49.5 (3)	O1A^iii^—Tl1A—O1A—Tl1A^iv^	−91.49 (15)
O1^i^—Tl1—O1—Tl1^i^	−8.2 (4)	O1A^iv^—Tl1A—O1A—Tl1A^iv^	−10.8 (5)
O1^ii^—Tl1—O1—Tl1^i^	−91.4 (5)	Tl1A^iii^—O1A—C1A—C2A^v^	68.4 (10)
Tl1^vii^—Tl1—O1—Tl1^i^	−50.1 (3)	Tl1A^iv^—O1A—C1A—C2A^v^	−171.6 (10)
Tl1^ii^—O1—C1—C2	−53 (2)	Tl1A—O1A—C1A—C2A^v^	−51.6 (10)
Tl1^i^—O1—C1—C2	66 (2)	Tl1A^iii^—O1A—C1A—C2A^vi^	−171.6 (10)
Tl1—O1—C1—C2	−174.2 (19)	Tl1A^iv^—O1A—C1A—C2A^vi^	−51.6 (10)
Tl1^ii^—O1—C1—C3	−173.5 (18)	Tl1A—O1A—C1A—C2A^vi^	68.4 (10)
Tl1^i^—O1—C1—C3	−55 (2)	Tl1A^iii^—O1A—C1A—C2A	−51.6 (10)
Tl1—O1—C1—C3	65 (2)	Tl1A^iv^—O1A—C1A—C2A	68.4 (10)
Tl1^ii^—O1—C1—C4	67.6 (19)	Tl1A—O1A—C1A—C2A	−171.6 (10)
Tl1^i^—O1—C1—C4	−173.7 (14)		

Symmetry codes: (i) −*x*+3/2, −*z*+3/2, *y*−1/2; (ii) −*x*+3/2, *z*+1/2, −*y*+3/2; (iii) −*x*+1, *y*, −*z*+1; (iv) −*x*+1, −*y*+1, *z*; (v) −*z*+1, −*x*+1, *y*; (vi) −*y*+1, *z*, −*x*+1; (vii) *x*, −*y*+2, −*z*+1.
